# Preoperative coronal alignment and tibial implant positioning affect condylar lift‐off in BCS‐TKA

**DOI:** 10.1002/jeo2.70609

**Published:** 2025-12-28

**Authors:** Kento Harada, Masayuki Kamimura, Yu Mori, Takashi Aki, Shunsuke Utsumi, Tomokazu Tanita, Toshiya Uehara, Toshimi Aizawa

**Affiliations:** ^1^ Department of Orthopaedic Surgery Tohoku University Graduate School of Medicine Sendai Japan

**Keywords:** arithmetic hip–knee–ankle angle, bi‐cruciate stabilized, condylar lift‐off, mechanical hip–knee–ankle angle, total knee arthroplasty

## Abstract

**Purpose:**

Condylar lift‐off is the phenomenon in which the femoral component lifts off from the polyethylene insert. In some cases, the mechanical hip–knee–ankle angle (mHKAA) appears more varus than the arithmetic hip–knee–ankle angle (aHKAA) in a standing position, which is thought to be related to lift‐off. This study analyzed factors influencing the difference between postoperative aHKAA and mHKAA in bi‐cruciate‐stabilized total knee arthroplasty (BCS‐TKA).

**Methods:**

This study included 100 knees that underwent BCS‐TKA. Full‐length standing radiographs of both lower extremities were obtained preoperatively and 1‐year postoperatively. The mechanical lateral distal femoral angle (mLDFA), mechanical medial proximal tibial angle (mMPTA), and mHKAA were measured and aHKAA (mMPTA−mLDFA) was calculated. The difference between postoperative aHKAA (post‐aHKAA) and post‐mHKAA was defined as post‐ΔHKAA (post‐mHKAA−post‐aHKAA). Correlations between post‐ΔHKAA and the measured parameters were analyzed, and multivariable linear regression analyses were performed to identify independent predictors of post‐ΔHKAA.

**Results:**

Post‐ΔHKAA was significantly correlated with preoperative mLDFA (pre‐mLDFA, *r* = −0.30, *p* < 0.001), pre‐aHKAA (*r* = 0.22, *p* = 0.02), pre‐mHKAA (*r* = 0.21, *p* = 0.03) and post‐mMPTA (*r* = −0.29, *p* = 0.004). Multivariable linear regression analyses revealed that pre‐mLDFA (*β* = −0.20, *p* = 0.02) and post‐mMPTA (*β* = −0.32, *p *= 0.01) were independently associated with post‐ΔHKAA. These results indicate that performing TKA in cases with severe femoral varus alignment and placing the tibial implant in valgus relative to the original joint surface leads to increased postoperative coronal varus alignment in the standing position.

**Conclusion:**

In BCS‐type TKA, postoperative condylar lift‐off in standing positions is more likely to occur in cases with severe preoperative femoral varus alignment or with valgus tibial implant placement.

**Level of Evidence:**

Level Ⅳ.

## Graphical abstract

Preoperative alignment and tibial implant position influence condylar lift‐off in BCS‐TKA.

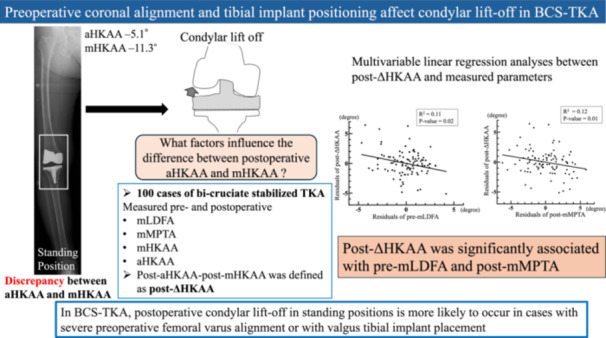

**Abbreviations**
ACL: anterior cruciate ligament
aHKAA: arithmetic hip‐knee‐ankle angle
BCS: bi‐cruciate stabilized
CR: cruciate‐retaining
CS: cruciate‐substituting
ICC: Intraclass correlation coefficient
LCL: lateral collateral ligament
mHKAA: mechanical hip‐knee‐ankle angle
mLDFA: mechanical lateral distal femoral angle
mMPTA: mechanical medial proximal tibial angle
OA: osteoarthritis
PCA: posterior condylar axis
PS: posterior‐stabilized
SEA: surgical condylar axis
TKA: total knee arthroplasty

## INTRODUCTION

Total knee arthroplasty (TKA) is a highly effective treatment for late‐stage osteoarthritis (OA), providing pain relief and improving daily activities [28]. Various types of TKA designs are now available, including cruciate‐retaining (CR), cruciate‐substituting (CS), posterior‐stabilized (PS) types, and others. The function of the anterior cruciate ligament (ACL) has recently been recognized as important for achieving good postoperative outcomes [29]. Among these multiple TKA designs, the bi‐cruciate stabilized (BCS) TKA design closely replicated the functions of the anterior and posterior cruciate ligaments in a normal knee joint [27]. Additionally, BCS‐TKA has demonstrated promising clinical outcomes concerning ACL functionality [12, 14, 22].

Condylar lift‐off refers to the phenomenon in which one femoral condyle loses contact with the insert, creating a gap on that side of the joint [26], and several studies have investigated the causes. Nilsson initially demonstrated that uneven bearing loads could lead to lift‐off due to asymmetric subsidence and tilting of the tibial component [24]. Lift‐off has also been associated with polyethylene wear [2] and implant loosening [9]. Therefore, knee surgeons must consider this phenomenon during preoperative planning to prevent complications.

Various methods for evaluating lift‐off in TKA have been reported in the literature. Using a computer simulation study, Kuriyama reported the relationship between femoral varus alignment, lateral collateral ligament (LCL) laxity, and lift‐off [16]. Sharma investigated the causes of lift‐off through fluoroscopic X‐ray imaging and analyzed the relationship between the range of knee motion and the frequency of lift‐off [25]. Hamai examined lift‐off using X‐ray photography under manual stress examination [7].

**Figure 1:**
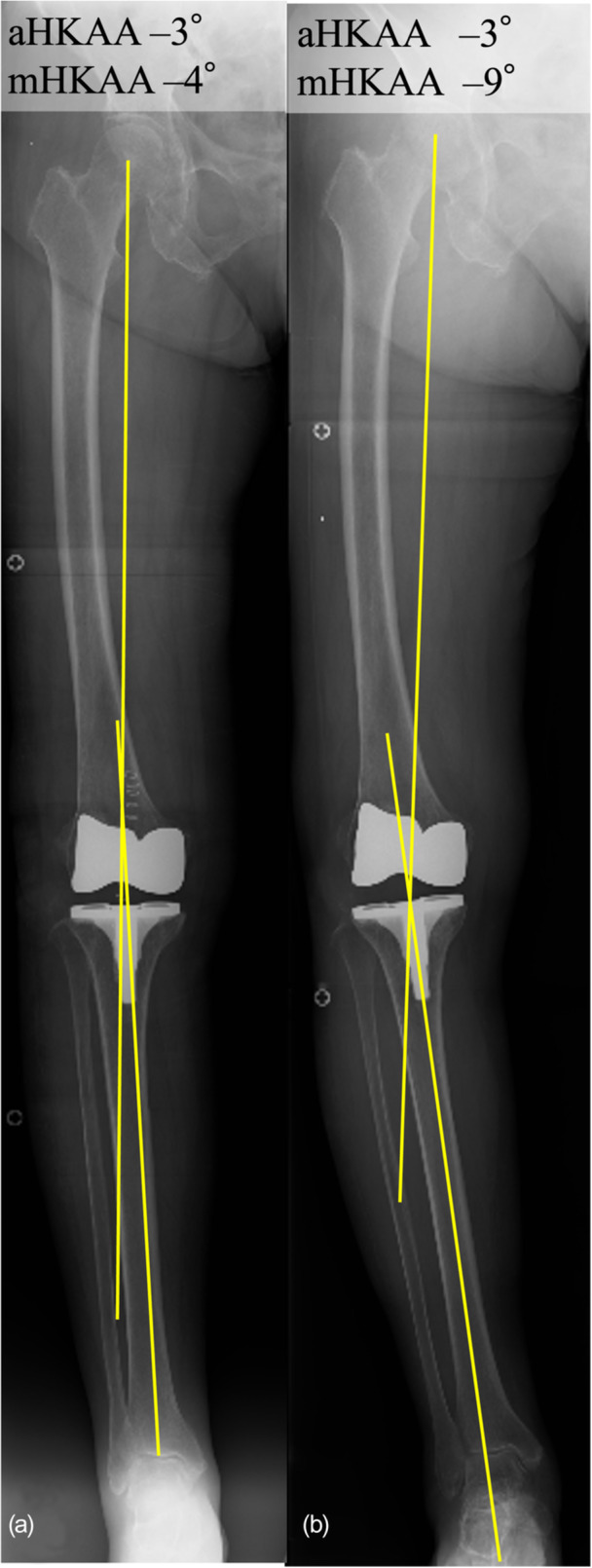
Differences between mechanical hip–knee–ankle angle (mHKAA) and arithmetic hip–knee–ankle angle (aHKAA) on the standing full‐length lower limb radiographs. While aHKAA is –3° and mHKAA is –4° in the supine position, the mHKAA showed greater varus alignment (–9°) in the standing position, indicating an increased discrepancy between mHKAA and aHKAA.

When evaluating alignment after TKA, it is sometimes observed that the mechanical hip–knee–ankle angle (mHKAA) appears more varus than the arithmetic hip–knee–ankle angle (aHKAA) in the standing position (Figure 1). In such cases, it can be inferred that lift‐off occurs in a standing position. A previous study has suggested that a greater correction of aHKAA is associated with increased postoperative lateral laxity assessed by stress radiographs [15], implying that coronal alignment assessment may serve as a valuable indicator for evaluating postoperative lateral laxity. To the best of our knowledge, there have been few reports on the relationship between condylar lift‐off in BCS‐TKA and coronal plane alignment, as assessed by radiographic examination. In this study, we hypothesized that the difference between postoperative mHKAA and aHKAA could quantitatively reflect the pathology of lift‐off. The primary aim of this study was to analyze the factors influencing the difference between postoperative aHKAA and mHKAA in patients undergoing BCS‐TKA.

## METHODS

### Participants

This study adhered to the ethical standards outlined in the Declaration of Helsinki and was approved by the Institutional Review Board of Tohoku University Hospital (approval number 2022‐1‐066). The study was conducted and reported in accordance with the STROBE (Strengthening the Reporting of Observational Studies in Epidemiology) guidelines. This retrospective study included patients with symptomatic OA undergoing BCS‐TKA (Journey II BCS; Smith & Nephew) at our institution from April 2017 to September 2021. The inclusion criteria included (1) patients in whom a BCS insert was used and (2) patients who underwent full‐length lower limb radiography both preoperatively and at 1‐year postoperatively. The exclusion criteria included (1) patients in whom a constrained insert was used, (2) patients with knee flexion contracture of more than 20 degrees before and after the operation, and (3) patients with a history of total hip or ankle arthroplasty, trauma or periarticular osteotomy around the knee. A total of 100 patients who met the above inclusion and exclusion criteria were selected and included in the final analysis.

### Surgical procedure

All surgeries were performed using the medial parapatellar approach by 10 surgeons at our hospital, following the same standardized protocol established at our institute. The distal femur and proximal tibia were cut using the intramedullary guide. Femoral and tibial coronal alignments were aimed at 90° to the mechanical axis. Since the Journey Ⅱ BCS insert incorporates a built‐in medial inclination, the postoperative joint line is not strictly perpendicular to the mechanical axis. Consequently, the final alignment tends to approximate anatomical alignment [20]. Flexion gap balancing was conducted using a modified gap technique [21]. Femoral component rotation was determined according to the surgical epicondylar axis (SEA), which is widely regarded as a reliable reference for rotational alignment in total knee arthroplasty [3]. Preoperatively, the angle between the posterior condylar axis (PCA) and the SEA was measured on preoperative imaging using planning software. Intraoperatively, a posterior referencing system was used to determine the femoral rotation angle. Tibial rotation was determined using one of two methods: the ROM technique [10] or Akagi's line [1], with the choice of method depending on the surgeon. Overall, the ROM technique was used in 52% of cases, whereas Akagi's line was applied in 48%. During surgery, the implant gap was measured using an independent medial‐lateral tensioner. A constrained BCS insert was utilized in case of medial instability at any flexion angles or severe lateral instability at extension (greater than 4 mm). All surgical planning was done using ZedKnee software (LEXI) or ZioCube (Ziosoft).

### Radiographic measurements

**Figure 2:**
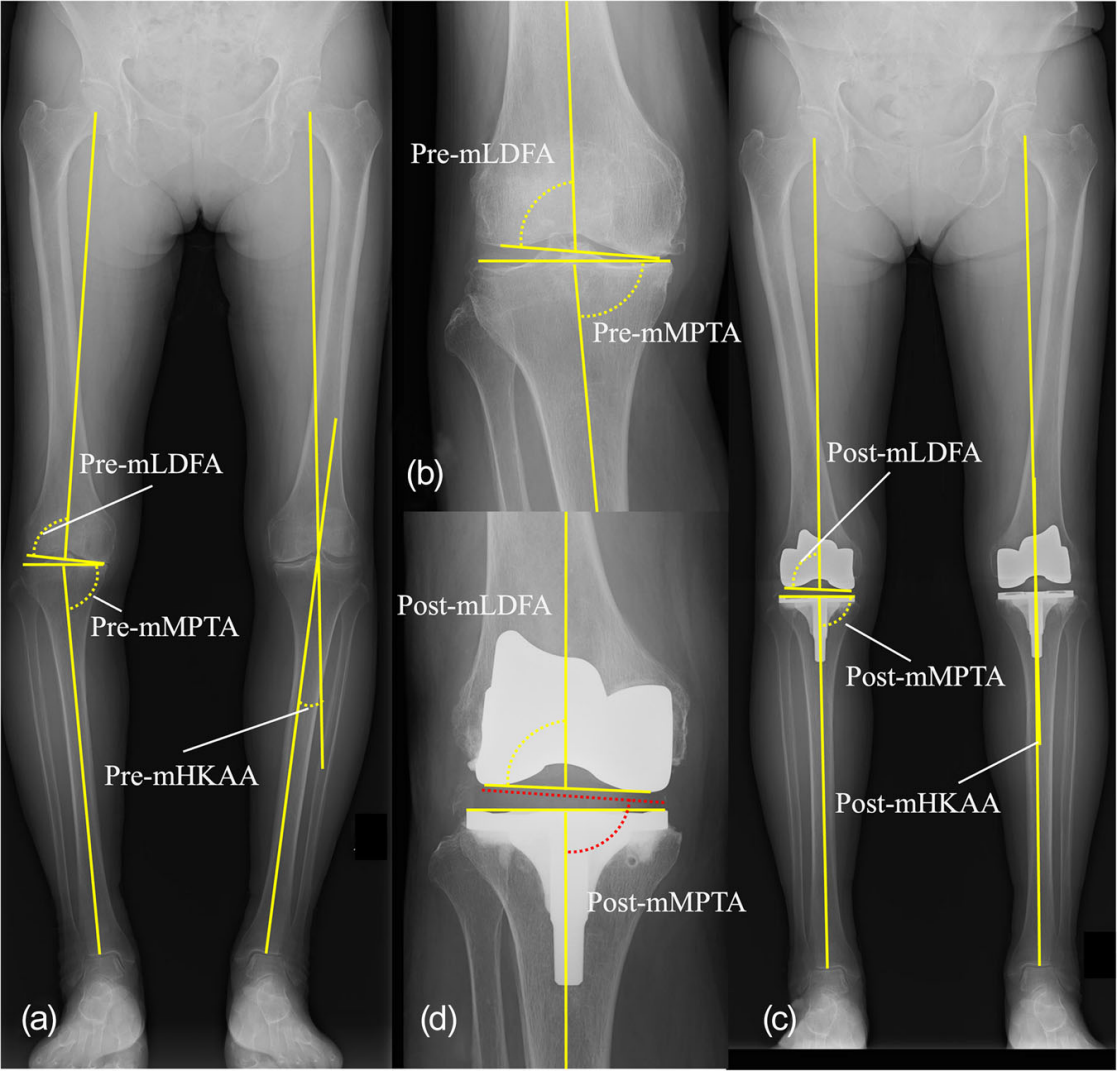
Measurement of pre‐ and postoperative parameters. mechanical lateral distal femoral angle (mLDFA), lateral angle between the femoral mechanical axis and distal femoral joint line (a, b); postoperatively, the distal femoral joint line was defined as the tangent to the distal surface of the femoral component (c, d). Mechanical medial proximal tibial angle (mMPTA), medial angle between tibial mechanical axis and proximal tibial joint line (a, b); postoperatively, proximal tibial line was defined as the tangent to the tibial component surface and adjusted for insert tilt calculated using the arctangent function (c, d). Mechanical hip–knee–ankle angle (mHKAA), angle between the femoral and tibial functional axes (a, c).

Plain radiographs were retrospectively assessed. Full‐length standing radiographs of both lower extremities were obtained preoperatively and 1‐year postoperatively to measure the alignment of the lower extremities. These radiographs covered the entire lower limb, including hip, knee and ankle joints. All images were acquired in a weight‐bearing position with the patella facing forward in a neutral rotational alignment, minimizing measurement errors due to limb malrotation, as described in a previously published protocol [19]. All radiographic measurements were performed using a digital measurement tool in the Picture Archiving and Communication System (Vue PACS, version 12.2.5.4000286; Carestream Health). The measurement parameters are shown in Figure 2. Preoperative and 1‐year postoperative mechanical lateral distal femoral angle (mLDFA), mechanical medial proximal tibial angle (mMPTA) and mHKAA were measured, and the aHKAA (mMPTA−mLDFA) was subsequently calculated. mLDFA was defined as the lateral angle between the femoral mechanical axis and the distal femoral joint line [8, 19]. In the postoperative assessment, the distal femoral joint line was defined as the tangent of the distal articular surface of the femoral component. mMPTA was defined as the medial angle between the tibial mechanical axis and the proximal tibia's joint line [8, 19]. Postoperatively, the proximal joint line was defined as the tangent to the surface of the tibial component. Since the polyethylene insert introduces a design‐dependent mediolateral inclination, the postoperative mMPTA was calculated by subtracting the insert tilt from this angle. The insert tilt was determined by first obtaining the mediolateral width specific to each implant size and then calculating the angle from the height difference between the medial and lateral sides of the tibial insert using the arctangent function. mHKAA is the angle formed by the femoral and tibial functional axis [18]. Preoperative values of each parameter were described with the prefix “pre‐” (e.g., pre‐mLDFA, pre‐mMPTA), and postoperative values were described with the prefix “post‐” (e.g., post‐mLDFA, post‐mMPTA).

Measurements were independently performed by two orthopedic surgeons at our hospital. Inter‐observer reliability was assessed using intraclass correlation coefficients (ICCs [1, 2]). The ICC values for measurements were as follows: pre‐mLDFA, 0.984 (95% confidence interval [CI], 0.972–0.990); pre‐mMPTA, 0.938 (95% CI, 0.898–0.960); pre‐mHKAA, 0.960 (95% CI, 0.897–0.988); post‐mLDFA, 0.922 (95% CI, 0.871–0.953); post‐mMPTA, 0.951 (95% CI, 0.890–0.983); and post‐mHKAA, 0.973 (95% CI, 0.950–0.986).

### Statistical analysis

Patients' demographic characteristics and measurement data are expressed as mean ± standard deviation (SD). Data normality was examined using the Shapiro–Wilk test. The difference between post‐aHKAA and post‐mHKAA was defined as post‐ΔHKAA (post‐mHKAA–post‐aHKAA); a negative post‐ΔHKAA indicated greater varus alignment in the standing position. The correlations between post‐ΔHKAA and each of the following parameters—pre‐mLDFA, pre‐mMPTA, pre‐mHKAA, pre‐aHKAA, post‐mLDFA and post‐mMPTA—were assessed using Pearson's correlation coefficient for parametric data and Spearman's rank correlation for nonparametric data through JMP 17 data analysis software (SAS Institute). The analysis was performed using R (version 4.2.2). A *p*‐value of <0.05 was considered statistically significant. Correlation coefficients were interpreted according to commonly accepted thresholds: <0.2 as very weak, 0.2–0.4 as weak, 0.4–0.7 as moderate and >0.7 as strong [23]. For variables that showed a statistically significant correlation with post‐ΔHKAA in the univariate analysis, multivariable linear regression analyses were performed with post‐ΔHKAA as the dependent variable. Age, sex, body mass index (BMI), surgeons and tibial rotation method were included as covariates to adjust for potential confounding effects.

## RESULTS

Patient characteristics in this study are shown in Table 1. The mean and SD values of the measured parameters are shown in Table 2. The preoperative measurements (mean ± SD) were as follows: mLDFA, 88.8 ± 2.8°; mMPTA, 84.7 ± 2.2°; mHKAA, −12.0 ± 5.8° and aHKAA, −4.2 ± 3.6°. The average postoperative measurements were: mLDFA, 87.8 ± 1.8°; mMPTA, 87.2 ± 1.8°; mHKAA, −1.8 ± 3.3° and aHKAA, −0.6 ± 2.6°. Post‐ΔHKAA was −1.2 ± 2.1°. The correlation between post‐ΔHKAA and measured parameters is shown in Table 3. In terms of the correlation between coronal alignments and post‐ΔHKAA, post‐ΔHKAA was correlated with pre‐mLDFA (*r* = −0.30, *p* < 0.001), pre‐aHKAA (*r* = 0.22, *p* = 0.02) and pre‐mHKAA (*r* = 0.21, *p* = 0.03) and post‐mMPTA (*r* = −0.29, *p* = 0.004). Multivariable linear regression analyses showed that pre‐mLDFA and post‐mMPTA were independently associated with post‐ΔHKAA. Pre‐mLDFA showed a significant negative association with post‐ΔHKAA (*β* = −0.217, 95% CI: −0.38 to −0.05, *p* = 0.011), indicating that greater preoperative femoral varus alignment was associated with an increased risk of postoperative lift‐off. Similarly, post‐mMPTA was negatively associated with post‐ΔHKAA (*β* = −0.389, 95% CI: −0.63 to −0.15, *p* = 0.002), suggesting that valgus positioning of the tibial component contributed to lift‐off. Other covariates, including age, sex, BMI, surgeon and tibial rotation method, were not significantly associated with post‐ΔHKAA.

**Table 1:** Patients characteristics (*N* = 100).

Demographics	Value
Mean Age (y/o) ± SD	75.0 ± 7.0
Sex (%)	Female: 80 (80.0%); male: 20 (20.0%)
Mean Height (cm) ± SD	152.8 ± 9.1
Mean body weight (kg) ± SD	64.7 ± 11.7
Mean BMI (kg/m^2^) ± SD	27.7 ± 4.0

Abbreviations: BMI, body mass index; SD, standard deviation.

**Table 2:** Mean ± SD and 95% CI values of pre‐ and postoperative mLDFA, mMPTA,mHKAA, aHKAA and ΔHKAA.

	Pre‐operative	95% CI	Post‐operative	95% CI	*p* value
mLDFA	88.8 ± 2.8°	88.3 to 89.4	87.8 ± 1.8°	87.4 to 88.2	<0.001
mMPTA	84.7 ± 2.2°	84.3 to 85.2	87.2 ± 1.8°	86.8 to 87.5	<0.001
mHKAA	–12.0 ± 5.8°	–13.1 to –10.8	–1.8 ± 3.3°	–2.47 to –1.17	<0.001
aHKAA	–4.2 ± 3.6°	–4.87 to –3.43	–0.6 ± 2.6°	–1.13 to –0.08	<0.001
Post‐ΔHKAA			–1.2 ± 2.1°	–1.64 to –0.79	

*Note*: ΔHKAA was calculated by postoperative mHKAA–postoperative aHKAA. A *p*‐value of <0.05 is considered significant.

Abbreviations: aHKAA, arithmetic hip‐knee‐ankle angle; CI, confidence interval; mLDFA, mechanical lateral distal femoral angle; mMPTA, mechanical medial proximal tibial angle; mHKAA, mechanical hip‐knee‐ankle angle; SD, standard deviation.

**Table 3:** Correlation between post‐ΔHKAA and measured parameters.

	*R* (95% CI)	*p* value
Pre‐mLDFA	–0.30 (–0.46 to –0.11)	<0.001
Pre‐mMPTA	0.001 (–0.20 to 0.20)	0.997
Pre‐mHKAA	0.21 (0.02 to 0.40)	0.035
Pre‐aHKAA	0.23 (0.03 to 0.41)	0.022
Post‐mLDFA	–0.17 (–0.36 to 0.02)	0.150
Post‐mMPTA	–0.29 (–0.46 to –0.10)	0.004

*Note*: A *p*‐value of <0.05 is considered significant.

Abbreviations: aHKAA, arithmetic hip‐knee‐ankle angle; CI, confidence interval; mLDFA, mechanical lateral distal femoral angle; mMPTA, mechanical medial proximal tibial angle; mHKAA, mechanical hip‐knee‐ankle angle; R, correlation coefficients.

## DISCUSSION

The most important findings of this study are that, in BCS‐TKA aiming at anatomical alignment, condylar lift‐off tends to increase when a preoperative femur exhibits a varus tendency. Additionally, lift‐off is exacerbated when the tibial implant is positioned in a valgus position. aHKAA is a relatively new concept that was recently proposed [5], and it remains unchanged between standing and lying positions. By comparing aHKAA with mHKAA, lift‐off can be quantitatively evaluated. To the best of our knowledge, this is the first study to investigate this relationship, making a significant contribution to the quantitative evaluation of lift‐off.

The ICC of mLDFA, mMPTA and mHKAA measurements in preoperative and postoperative surgery was greater than or equal to 0.90, demonstrating a high level of reliability. Previous studies have reported ICC values of 0.90 or higher for MPTA and LDFA [4], indicating strong consistency, and our findings align with those in the previous literature.

**Figure 3:**
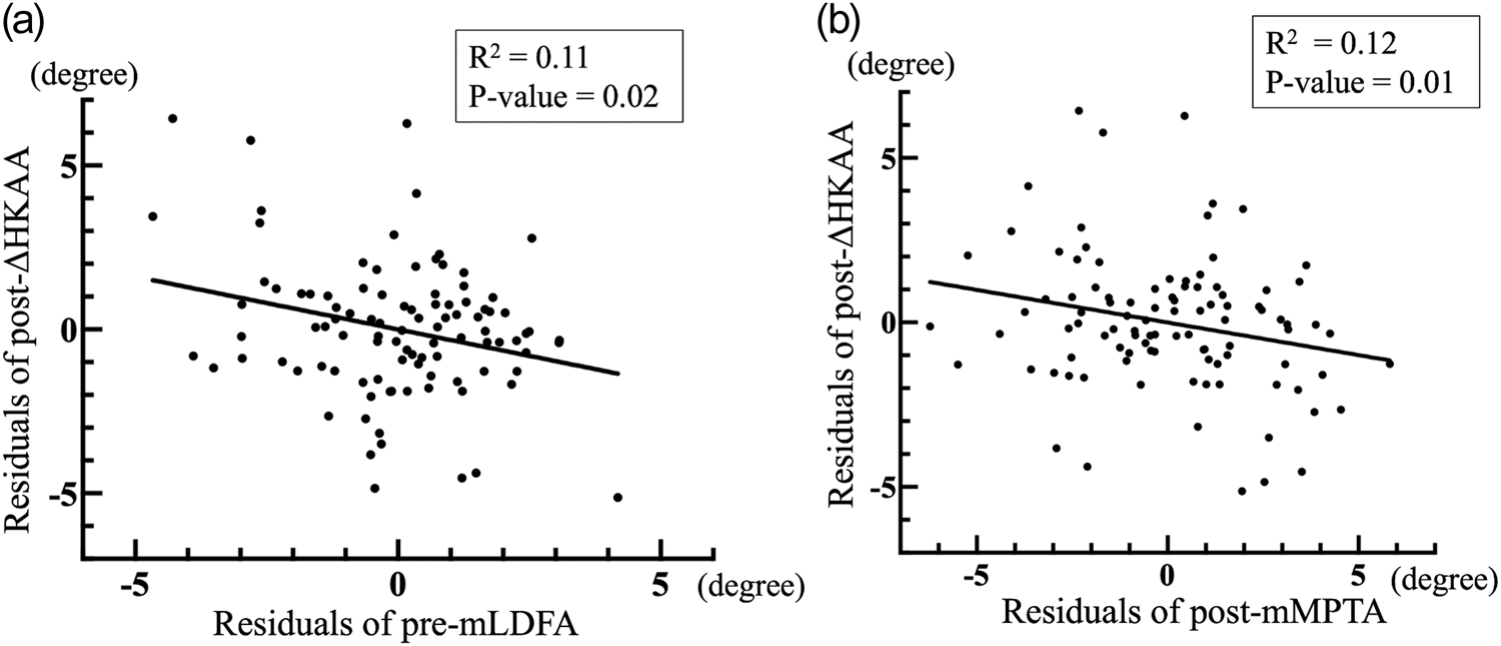
Multivariable linear regression analyses for factors associated with post‐ΔHKAA. Pre‐mLDFA (*β* = –0.22, *p* = 0.011; *R*² = 0.11, Figure a) and post‐mMPTA (*β* = –0.39, *p* = 0.002; *R*² = 0.12, Figure b) were independently and negatively associated with post‐ΔHKAA. HKAA, hip–knee–ankle angle; mLDFA, mechanical lateral distal femoral angle.

In this study, post‐ΔHKAA was defined as the difference between post‐mHKAA and post‐aHKAA, with negative values indicating relatively greater varus alignment in the standing position. Regarding the association between preoperative coronal alignment and lift‐off, a multivariable linear regression analysis revealed that pre‐mLDFA was independently associated with post‐ΔHKAA (Table 4 and Figure 3a). When interpreted quantitatively, each 1° increase in pre‐mLDFA resulted in an approximate 0.22° decrease in ΔHKAA. This finding indicates that greater preoperative femoral varus alignment remains a significant predictor of postoperative lift‐off. This result also suggests that anatomically aligned TKA in cases with severe femoral varus may be associated with a higher risk of postoperative lift‐off. When the distal femur exhibits a pronounced varus alignment, performing a distal femoral cut perpendicular to the mechanical axis results in a greater resection of the lateral distal femoral condyle. In the Journey II BCS, the distal thickness of the femoral implant is 9.5 mm medially and 7.0 mm laterally. Therefore, a mismatch occurs between the thickness of the bone cut and the implant at the lateral distal femur, which is considered to contribute to postoperative lateral ligamentous laxity. Post‐ΔHKAA was correlated with pre‐mLDFA, whereas no correlation was observed with post‐mLDFA. Kuriyama reported that in computer simulation models, condylar lift‐off occurred easily in the stance phase in femoral varus alignments greater than 3° with slight lateral slack, indicating the importance of neutral alignment [16]. The finding in the present study that no association was observed between post‐mLDFA and post‐ΔHKAA suggests that the postoperative femoral implant placement may not be related to condylar lift‐off, which contrasts with the previous report [16]. Only one out of 100 cases exhibited femoral varus alignment greater than 3° postoperatively in this series, which may account for the discrepancy in findings. Concerning the relationship between postoperative coronal alignment and lift‐off, a multivariable linear regression analysis revealed that post‐mMPTA and post‐ΔHKAA (Table 4 and Figure 3b) are significantly associated. Quantitatively, each 1° increase in post‐mMPTA was associated with an approximately 0.39° decrease in ΔHKAA, indicating that valgus positioning of the tibial component is an independent predictor of postoperative lift‐off. It has been reported that in TKA, changes in MPTA affect the knee ligament balance, especially when enhanced tibial varus alignment leads to increased medial loosening in both knee extension and flexion positions [6]. Innocenti reported that valgus positioning of the tibial implant reduces the strain on the LCL [11]. A proximal tibial cut in valgus results in greater resection on the lateral side compared with the medial side. A mismatch between the resected bone and implant thickness at the lateral proximal tibia is a potential cause of lateral ligamentous laxity.

**Table 4:** Multivariable linear regression analysis for predictors of post‐ΔHKAA, adjusted for age, sex, BMI, surgeon and tibial rotational technique.

	*β* (95% CI)	SE	*t* value	*p* value
Age	–0.05 (–0.11 to 0.01)	0.031	–1.63	0.106
Sex	–0.25 (–1.33 to 0.84)	0.548	–0.45	0.654
BMI	–0.07 (–0.18 to 0.04)	0.054	–1.3	0.196
Surgeons	–0.02 (–0.18 to 0.14)	0.081	–0.27	0.791
Tibial rotational technique	0.11 (–0.76 to 0.98)	0.437	0.25	0.806
Pre‐mLDFA	–0.20 (–0.37 to –0.03)	0.086	–2.3	0.024
Pre‐mHKAA	0.06 (–0.02 to 0.14)	0.04	1.54	0.698
Pre‐aHKAA	0.11 (–0.18 to 0.04)	0.062	1.77	0.080
Post‐mMPTA	–0.32 (–0.56 to –0.08)	0.122	–2.65	0.010

*Note*: A *p* value of <0.05 is considered significant.

Abbreviations: aHKAA, arithmetic hip–knee–ankle angle; BMI, body mass index; CI, confidence interval; mLDFA, mechanical lateral distal femoral angle; mMPTA, mechanical medial proximal tibial angle; mHKAA, mechanical hip–knee–ankle angle; SE, standard error; β, regression coefficient.

There have been many reports about the causes of lift‐off. Insall reported that malalignment of the femoral component in PS‐TKA may lead to lift‐off and mentioned the importance of medial‐lateral ligament balance during flexion [13]. Previous reports on implant designs have indicated that lift‐off is more likely to occur with PS implants [2, 17]. Hamai reported that lift‐off is less likely to occur in well‐balanced TKA [7], emphasizing the importance of achieving balanced medial and lateral ligament tension during this procedure. These reports suggest that lift‐off arises from multiple factors, including implant designs, coronal and rotational implant alignment, intraoperative knee joint balance and others. Since postoperative lift‐off negatively affects patients' satisfaction and implant survival [2, 9], surgeons should develop preoperative surgical plans to prevent this phenomenon. Our study found that valgus positioning of the tibial implant was associated with postoperative lift‐off. Combined with the results of the previous report, which emphasized the importance of femoral neutral alignment in preventing lift‐off [16], these results suggest that maintaining neutral alignment for both the femur and tibia may help reduce the frequency of lift‐off.

Although ΔHKAA was used as a surrogate quantitative marker for lift‐off in this study, a clinically validated threshold value has not yet been established in the literature. Therefore, we analyzed ΔHKAA as a continuous variable, with the understanding that larger ΔHKAA values may reflect greater coronal imbalance. Future studies correlating ΔHKAA with dynamic assessments such as stress radiographs or fluoroscopic analysis will be essential to validate its clinical significance and to define a meaningful cutoff value that can be applied in daily clinical practice.

### Limitations

This study has several limitations. First, this study included only patients who received BCS‐type implants, and the target alignment was limited to anatomical alignment. Therefore, both the implant type and alignment approach were restricted. It remains unclear whether a similar phenomenon would occur with other implant designs and alignment goals, such as kinematic alignment. Second, it is unclear whether the lift‐off evaluated using ΔHKAA, as measured from this study's radiograph, is comparable to the results obtained through stress radiography or fluoroscopic imaging in other studies. Therefore, it is necessary to assess lift‐off using multiple methods, such as X‐ray imaging and stress radiography. Moreover, ΔHKAA may be influenced by factors other than coronal implant alignment, including intraoperative soft‐tissue balance, collateral ligament tension, joint line position and other surgical variables. These factors were not directly evaluated in the present study and should be considered in future research to gain a comprehensive understanding of the multifactorial mechanisms contributing to lift‐off. Third, this study did not evaluate differences in clinical scores and postoperative functions of the knee joints between patients with and without lift‐off. Further investigation is needed to determine the impact of this phenomenon on postoperative clinical outcomes. Finally, although some correlations reached statistical significance, their magnitude was weak. This suggests that the strength of association between preoperative alignment and postoperative lift‐off may be limited, and the clinical relevance of these findings should be interpreted with caution.

## CONCLUSION

In BCS‐type TKA with femoral and tibial cuts made perpendicular to the functional axis, postoperative condylar lift‐off was likely to be observed in cases with severe preoperative femoral varus deformity, as well as in cases with valgus placement of the tibial implant. As the condylar lift‐off phenomenon negatively affects patients' satisfaction and implant survival, further investigation into the factors contributing to lift‐off is essential to improve postoperative outcomes.

## AUTHOR CONTRIBUTIONS

All authors are responsible for the work described in this article. Kento Harada, Masayuki Kamimura, Yu Mori, Takashi Aki, Shunsuke Utsumi, Tomokazu Tanita, Toshiya Uehara and Toshimi Aizawa were involved in the conception, design, and planning of the study. Kento Harada, Masayuki Kamimura, and Yu Mori were involved in the data measurement and analysis. Kento Harada, Masayuki Kamimura, Yu Mori, Takashi Aki, Shunsuke Utsumi, Tomokazu Tanita, Toshiya Uehara and Toshimi Aizawa interpreted the study results. All authors contributed to the critical review and approved the final manuscript.

## CONFLICT OF INTEREST STATEMENT

The authors declare no conflicts of interest.

## ETHICS STATEMENT

This retrospective study was conducted following the ethical standards outlined in the Declaration of Helsinki and was approved by the Institutional Review Board of Tohoku University Hospital (approval number 2022‐1‐066).

## Data Availability

The authors have nothing to report.
